# Associations Between Perceived Discrimination, Screening Mammography, and Breast Cancer Stage at Diagnosis: A Prospective Cohort Analysis

**DOI:** 10.1245/s10434-024-15757-0

**Published:** 2024-07-26

**Authors:** Alexandra E. Hernandez, Peter A. Borowsky, Maya Lubarsky, Carin Carroll, Seraphina Choi, Susan Kesmodel, Michael Antoni, Neha Goel

**Affiliations:** 1 Division of Surgical Oncology, Department of Surgery, Sylvester Comprehensive Cancer Center, University of Miami Miller School of Medicine, Miami, FL USA; 2 Sylvester Comprehensive Cancer Center, University of Miami, Miami, FL USA; 3 University of Miami Miller School of Medicine, Miami, FL USA; 4 Department of Human Genetics, University of Miami Miller School of Medicine, Miami, FL USA; 5 Department of Psychology, University of Miami Miller School of Medicine, Miami, FL USA

**Keywords:** Breast cancer, Disparities, Structural racism, Perceived discrimination, Stage at diagnosis, Screening mammography

## Abstract

**Background:**

Despite higher breast cancer screening rates, black women still are more likely to have late-stage disease diagnosed. This disparity is influenced in part by structural and interpersonal racism. This prospective study sought to determine how interpersonal factors, including perceived discrimination, influence screening and stage of disease at diagnosis.

**Methods:**

A prospective cohort study analyzed adult women with stages I to IV breast cancer from the Miami Breast Cancer Disparities Study. Perceived discrimination and mistrust of providers were assessed using previously validated questionnaires. Multivariable logistic regression was used to evaluate the odds of screening mammography utilization and late-stage breast cancer at diagnosis.

**Results:**

The study enrolled 342 patients (54.4 % Hispanic, 15.8 % white, and 17.3 % black). Multivariate regression, after control for both individual- and neighborhood-level factors, showed that a higher level of perceived discrimination was associated with greater odds of late-stage disease (adjusted odds ratio [aOR], 1.06; range, 1.01–1.12); *p* = 0.022) and lower odds of screening mammography (aOR, 0.96; range, 0.92–0.99; *p* = 0.046). A higher level of perceived discrimination also was negatively correlated with multiple measures of provider trust.

**Discussion:**

This study identified that high perceived level of discrimination is associated with decreased odds of ever having a screening mammogram and increased odds of late-stage disease. Efforts are needed to reach women who experience perceived discrimination and to improve the patient–provider trust relationship because these may be modifiable risk factors for barriers to screening and late-stage disease presentation, which ultimately have an impact on breast cancer survival.

Although recent advances in screening, diagnosis, and treatment have reduced breast cancer mortality, still, socioeconomic, racial, and ethnic disparities persist.^1,2^ Although black women have higher breast cancer screening rates than white women, black women still are more likely to receive a diagnosis of late-stage breast cancer.^3–5^ One important factor leading to these disparities is structural racism.^1,6^ Structural racism refers to “the totality of ways in which societies foster racial discrimination via mutually reinforcing inequitable systems that in turn reinforce discriminatory beliefs, values, and distribution of resources.”^7^ The consequences of such structural racism shape access to care and health outcomes.

The effects of structural racism on our health care system trickle into individual patient and provider behaviors, affecting patient trust and outcomes.^8–10^ Perceived discrimination faced by patients can alter or affect health behaviors and have a negative effect on many aspects of a person’s health.^11–13^ Discrimination is defined as unequal treatment of individuals based on certain characteristics such as race, ethnicity, or religion. Perceived discrimination affects mental health, including depression, anxiety, and psychological stress.^11^ The perception of discrimination has been shown to prevent people from going to places at which they perceive discrimination to occur, including hospitals and clinical care centers.^14^

Research has shown that racial discrimination is associated with multiple indicators of adverse cardiovascular disease outcomes, incidence of obesity, hypertension, and poorer general health.^8,9,11^ Other studies have shown that discriminated groups are more likely than non-discriminated groups to refrain from seeking medical attention or to underutilize medical services.^15^

In the Black Women’s Health Study,^16^ perceived discrimination has been associated with higher incidence breast cancer among black women. However, evidence for the effects of perceived discrimination on screening behavior is mixed.^16–19^ There remains a gap in the literature assessing the association of perceived discrimination with screening and stage of disease at presentation among breast cancer patients. Therefore, we sought to determine whether perceived discrimination is predictive of lower screening mammography utilization and late breast cancer stage at diagnosis (stages III or IV vs I or II) in a socioeconomically and racially and ethnically diverse South Florida population. We additionally sought to analyze the associations between perceived discrimination and provider mistrust based on patient-reported survey items.

## Materials and Methods

### Study Design, Patient Population, and Survey

We conducted a prospective cohort study of stages I to IV breast cancer patients enrolled in the Miami Breast Cancer Disparities Study protocol. All English, Spanish, and Haitian Creole-speaking women 18 years of age or older from Miami-Dade County with a diagnosis of stages I to IV breast cancer were eligible for inclusion. Patients unable to consent were excluded. Informed consent was obtained from all the subjects involved in the study. The study participants were administered a survey of questions, which included sociodemographic and medical information as well as validated scales to measure social determinants of health. This study was approved by the institutional review board of the University of Miami and Jackson Health System.

### Perceived Discrimination

To measure perceived discrimination, we used the Expanded Everyday Discrimination Scale (EEDS), a validated scale that assesses chronic and unfair treatment in everyday life.^7^ The scale consists of the following 10 questions:

1. You are treated with less courtesy than other people are.
2. You are treated with less respect than other people are.
3. You receive poorer service than other people at restaurants or stores
4. People act as if they think you are not smart.
5. People act as if they are afraid of you.
6. People act as if they think you are dishonest.
7. People act as if they are better than you are.
8. You are called names or insulted.
9. You are threatened or harassed.
10. You are followed around in stores.

In response to each statement, the participants were asked to choose from the following responses: “never,” “less than once a year,” “a few times a year,” “a few times a month,” “at least once a week,” and “almost every day.”^20^ Patients who reported any degree of perceived discrimination in this survey were asked the follow-up question: “What do you think is the main reason for these experiences?”

Based on prior research, the 10 questions in the scale were coded by assigning a score from 0 (never experienced) to 3 (experienced at least a few times a month).^21^ Each participant’s composite score was calculated by the sum of the scores from the 10 questions. The composite score ranged from 0 (least perceived discrimination) to 30 (most perceived discrimination).

### Patient–Provider Mistrust

The patients also were administered surveys to assess their trust and comfortability with their provider. This was measured using a questionnaire with the following six questions:

1. You understand everything your doctor tells you.
2. You feel comfortable asking your doctor questions.
3. You feel comfortable telling your doctor when you don’t understand something.
4. You feel comfortable telling your doctor when you don’t want a test or procedure.
5. You believe your doctor has your best interests at heart.
6. You trust your doctor.

The answer choices were “strongly agree,” “agree,” “strongly disagree,” and “disagree,” and the questionnaires were scored on a Likert scale.

### Covariables

Patient characteristics, collected by both survey response and electronic medical review, included age, self-reported race and ethnicity, preferred language, annual household income, employment status, education level, and insurance status. Patient race was categorized as “white,” “black,” or “other/not recorded/missing, and ethnicity was categorized as Hispanic or non-Hispanic based on the survey response. The covariables included in the multivariate regression models were race and ethnicity, annual household income, Area Deprivation Index (ADI), education level, employment, and receipt of annual screening mammograms after the age of 40 years (applicable only in the model predicting stage at diagnosis). Individual annual household income was categorized into quartiles (<$15,999, $16,000–$49,999, $50,000–$99,999, >$99,999).

The ADI is a validated, multidimensional measure of neighborhood disadvantage. It allows for rankings of neighborhoods by socioeconomic disadvantage in a region of interest (e.g., at the state or national level). We used the state-level ADI in our analysis, which is provided in deciles from 1 to 10 and treated as a continuous variable.

Education level was categorized as either college graduate or lower education (some college or technical school, high school graduate, some high school, and elementary school). Employment was categorized as employed versus unemployed.

### Outcomes

Our primary outcome was American Joint Committee on Cancer (AJCC) eighth-edition clinical stage at diagnosis, categorized as early (stages I and II) versus late (stages III and IV). This was collected from patient electronic medical records. Receipt of annual screening mammograms after age 40 years (categorized as yes or no) was assessed in the patient survey and verified on electronic medical records. Patients underwent screening mammography either within our hospital system or at outside facilities. Mammograms obtained outside our system were sent to our institution and subsequently uploaded into our electronic medical records.

### Statistical Analysis

For categorical variables, the chi-square test was used to compare individual variables by stage at diagnosis and screening mammography. For continuous variables, normality of the data was assessed using the Shapiro-Wilk test. Depending on the normality results, either parametric (*t* test) or non-parametric (Mann–Whitney *U* test) analyses were performed accordingly. Cronbach’s alpha was used to measure survey reliability for the mistrust surveys in our patient population. Spearman’s correlation tests were used to assess and describe the strength of correlation between EEDS score and patient–provider measures of mistrust. To evaluate the odds of late-stage versus early-stage disease at diagnosis as well as receipt of screening mammography (yes vs no), uni- and multivariable logistic regression analyses were performed to control for potential confounders. All analyses were performed using IBM SPSS Statistics version 28.0.0.0 (SPSS, Chicago, IL).

## Results

### Patient and Tumor Characteristics and Stage at Diagnosis

Of the 570 breast cancer patients in our prospective cohort, 342 had complete survey data, disease stage at diagnosis, and screening mammography information. In terms of race and ethnicity, 15.8 % of the patients (*n* = 54) were non-Hispanic white, 17.3 % (*n* = 59) were non-Hispanic black, and 54.4 % (*n* = 186) were Hispanic. The mean age at diagnosis differed significantly between the patients who had undergone screening mammography (mean, 57.68 ± 9.5 years) and those who had not (mean, 48.01 ± 13.8 years) (*p* < 0.001. Race and ethnicity were significantly associated with the disease stage at diagnosis (*p* = 0.012). White patients comprised 24.1 % of late-stage diagnoses versus 14.2 % of early-stage diagnoses, whereas the Hispanic patients comprised 35.2 % of late-stage diagnoses, lower than their 58.3 % representation of early-stage diagnoses.

No significant association was found between race/ethnicity and screening mammography (*p* = 0.687). The patients with a late-stage diagnosis had a higher mean ADI (mean, 5 ± 3) than those with an early-stage diagnosis (mean, 4 ± 3) (*p* = 0.019). Similarly, those who had not undergone screening mammography had a higher mean ADI (mean, 5 ± 3) than those who had (mean, 4 ± 3) (*p* = 0.013; Table 1).

**Table 1:** Individual- and contextual-level characteristics by breast cancer stage at diagnosis and screening mammography utilization

	Stage at diagnosis			Screening mammography		
	Early stage	Late stage	*p* Value	Yes	No	*p* Value
	*n* = 288 *n* (%)	*n* = 54 *n* (%)		*n* = 259 *n* (%)	*n* = 83 *n* (%)	
Mean age at diagnosis (years)	55.84 ± 11.23	52.72 ± 12.41	0.07	57.68 ± 9.5	48.01 ± 13.8	<0.001
Race/ethnicity			0.012			0.687
White	41 (14.2)	13 (24.1)		38 (14.7)	16 (19.3)	
Black	48 (16.7)	11 (20.4)		47 (18.1)	12 (14.5)	
Hispanic	168 (58.3)	19 (35.2)		143 (55.2)	44 (53.0)	
Other/not recorded/missing	7 (2.4)	2 (3.7)		31 (12.0)	11 (13.3)	
Preferred language			0.696			0.462
English	159 (55.2)	32 (59.3)		141 (54.4)	50 (60.2)	
Spanish	122 (42.4)	20 (37.0)		112 (43.2)	30 (36.1)	
Haitian Creole	7 (2.4)	2 (3.7)		6 (2.3)	3 (3.6)	
Annual household income ($)			0.358			0.555
<15,999	57 (23.5)	15 (34.1)		54 (25.0)	18 (25.4)	
$16,000–49,999	71 (29.2)	14 (31.8)		60 (27.8)	25 (35.2)	
50,000–99,999	64 (26.3)	8 (18.2)		55 (25.5)	17 (23.9)	
>99,999	51 (21.0)	7 (15.9)		47 (21.8)	11 (15.5)	
Employment status			0.316			0.425
Employed	152 (52.8)	24 (45.3)		130 (50.4)	46 (55.4)	
Unemployed	136 (47.2)	29 (54.7)		128 (49.6)	37 (44.6)	
Education level			0.055			0.461
College graduate	199 (74.5)	30 (61.2)		175 (73.5)	54 (69.2)	
Some college or technical school, high school graduate, some high school	68 (25.5)	19 (38.8)		63 (26.5)	24 (30.8)	
Insurance status			0.253			0.215
Private	132 (45.8)	20 (37.0)		115 (44.4)	37 (44.6)	
Medicare	44 (15.3)	7 (13.0)		43 (16.6)	8 (9.6)	
Medicaid or other government insurance	88 (30.6)	24 (44.4)		84 (32.4)	28 (33.7)	
Uninsured, self-pay, NOS	24 (8.3)	3 (5.6)		17 (6.6)	10 (12.0)	
Mean Area Deprivation Index	4 ± 3	5 ± 3	0.019	4 ± 3	5 ± 3	0.013
Median EEDS composite score (IQR)	1 (0–6)	2 (0–10)	0.034	1 (0–6)	2 (0–9)	0.021

NOS, not otherwise specified; EEDS, Expanded Everyday Discrimination Scale; IQR, interquartile range

A higher median EEDS composite score was observed for the patients with a late-stage diagnosis (median, 2; interquartile range [IQR], 0–10) than those with an early-stage diagnosis (median 1; IQR, 0–6) (*p* = 0.034). The patients without screening mammography also had a higher median EEDS composite score (median, 2; IQR, 0–9) than those with screening (median, 1; IQR, 0–6) (*p* = 0.021.

In contrast, factors such as the preferred language, annual household income, employment status, education level, and insurance status did not demonstrate statistically significant associations with the disease stage at diagnosis or the use of screening mammography.

### EEDs and Screening Mammography

The EEDS questions that showed a statistically significant association with the use of screening mammography were “You are followed around in stores” (patients with screening vs those without screening: never [89.6 % vs 78.3 %], less than once a year [5.0 % vs 10.8 %], a few times a year [4.2 % vs 6.0 %], at least a few times a month [1.2 % vs 4.8 %]; *p* = 0.004); “You are called names or insulted” (patients with screening vs those without screening: never [88.4 % vs 71.1 %], less than once a year [6.2 % vs 19.3 %], a few times a year [1.9 % vs 3.6 %}, at least a few times a month [3.5 % vs 6.0 %]; *p* = 0.001); and “You are threatened or harassed” (patients with screening vs those without screening: never [92.6 % vs 79.5 %], less than once a year [3.9 % vs 14.5 %], a few times a year [2.3 % vs 3.6 %]; *p* = 0.032). Furthermore, the individuals with screening mammography were less likely to report that they had been threatened or harassed (never [92.6 % vs 79.5 %], less than once a year [3.9 % vs 14.5 %; *p* = 0.032; Table 2).

**Table 2:** Level of perceived discrimination using Expanded Everyday Discrimination Scale (EEDS) responses by breast cancer stage at diagnosis and screening mammography utilization

	Stage at diagnosis			Screening mammography		
	Early stage	Late stage	*p* Value	Yes	No	*p* Value
	*n* = 288 *n* (%)	*n* = 54 *n* (%)		*n* = 259 *n* (%)	*n* = 83 *n* (%)	
You are treated with less courtesy than other people are			0.727			0.608
Never	190 (66)	32 (59.3)		173 (66.8)	49 (59.0)	
Less than once a year	42 (14.6)	10 (18.5)		38 (14.7)	14 (16.9)	
A few times a year	33 (11.5)	6 (11.1)		28 (10.8)	11 (13.3)	
At least a few times a month	23 (8)	6 (11.1)		20 (7.7)	9 (10.8)	
You are treated with less respect than other people are			0.284			0.559
Never	197 (68.4)	31 (57.4)		178 (68.7)	50 (60.2)	
Less than once a year	49 (17)	11 (20.4)		43 (16.6)	17 (20.5)	
A few times a year	20 (6.9)	4 (7.4)		17 (6.6)	7 (8.4)	
At least a few times a month	22 (7.6)	8 (14.8)		21 (8.1)	9 (10.8)	
You receive poorer service than other people at restaurants or stores			0.009			0.18
Never	210 (72.9)	30 (55.6)		189 (73.0)	51 (61.4)	
Less than once a year	44 (15.3)	10 (18.5)		39 (15.1)	15 (18.1)	
A few times a year	17 (5.9)	10 (18.5)		17 (6.6)	10 (12.0)	
At least a few times a month	17 (5.9)	4 (7.4)		14 (5.4)	7 (8.4)	
People act as if they think you are not smart			0.334			0.14
Never	200 (69.4)	34 (63)		183 (70.7)	51 (61.4)	
Less than once a year	42 (14.6)	8 (14.8)		37 (14.3)	13 (15.7)	
A few times a year	25 (8.7)	4 (7.4)		22 (8.5)	7 (8.4)	
At least a few times a month	21 (7.3)	8 (14.8)		17 (6.6)	12 (14.5)	
People act as if they are afraid of you			0.026			0.365
Never	257 (89.2)	41 (75.9)		230 (88.8)	68 (81.9)	
Less than once a year	17 (5.9)	5 (9.3)		15 (5.8)	7 (8.4)	
A few times a year	7 (2.4)	3 (5.6)		7 (2.7)	3 (3.6)	
At least a few times a month	7 (2.4)	5 (9.3)		7 (2.7)	5 (6.0)	
People act as if they think you are dishonest			0.403			0.064
Never	254 (88.2)	43 (79.6)		230 (88.8)	67 (80.7)	
Less than once a year	19 (6.6)	6 (11.1)		16 (6.2)	9 (10.8)	
A few times a year	6 (2.1)	2 (3.7)		7 (2.7)	1 (1.2)	
At least a few times a month	9 (3.1)	3 (5.6)		6 (2.3)	6 (7.2)	
People act as if they're better than you are			0.092			0.547
Never	190 (66)	26 (48.1)		169 (65.3)	47 (56.6)	
Less than once a year	39 (13.5)	11 (20.4)		35 (13.5)	15 (18.1)	
A few times a year	38 (13.2)	10 (18.5)		35 (13.5)	13 (15.7)	
At least a few times a month	21 (7.3)	7 (13)		20 (7.7)	8 (9.6)	
You are called names or insulted			0.015			0.001
Never	250 (86.8)	38 (70.4)		229 (88.4)	59 (71.1)	
Less than once a year	24 (8.3)	8 (14.8)		16 (6.2)	16 (19.3)	
A few times a year	5 (1.7)	3 (5.6)		5 (1.9)	3 (3.6)	
At least a few times a month	9 (3.1)	5 (9.3)		9 (3.5)	5 (6.0)	
You are threatened or harassed			0.008			0.032
Never	259 (90.2)	46 (85.22)		239 (92.6)	66 (79.5)	
Less than once a year	19 (6.6)	3 (5.6)		10 (3.9)	12 (14.5)	
A few times a year	4 (1.4)	5 (9.3)		6 (2.3)	3 (3.6)	
At least a few times a month	5 (1.7)	0 (0)		3 (1.2)	2 (2.4)	
You are followed around in stores			0.042			0.004
Never	256 (88.9)	41 (75.9)		232 (89.6)	65 (78.3)	
Less than once a year	17 (5.9)	5 (9.3)		13 (5.0)	9 (10.8)	
A few times a year	10 (3.5)	6 (11.1)		11 (4.2)	5 (6.0)	
At least a few times a month	5 (1.7)	2 (3.7)		3 (1.2)	4 (4.8)	

Multivariable logistic regression showed that a high level of perceived discrimination was associated with never having had a screening mammogram (adjusted odds ratio [aOR], 0.956; 95 % confidence interval [CI], 0.917–0.997; *p* = 0.046) after control for individual- and contextual-level factors (Table 3).

**Table 3:** Multivariable logistic regression for screening mammography utilization and clinical stage at diagnosis.

	Screening mammography		Late stage at diagnosis	
	aOR (95 % CI)	*p* Value	aOR (95 % CI)	*p* Value
Level of perceived discrimination (composite continuous)	0.956 (0.917–0.997)	0.046	1.062 (1.009–1.118)	0.022
Race/ethnicity (vs non-Hispanic white)				
Non-Hispanic black	2.065 (0.81–5.264)	0.129	0.945 (0.335–2.667)	0.915
Hispanic	1.615 (0.759–3.435)	1.615	0.288 (0.108–0.768)	0.013
Other	1.888 (0.68–5.24)	0.222	1.425 (0.108–0.768)	0.537
Annual household income ($) (quartiles vs >100,000)				
<15,999	0.889 (0.341–2.322)	0.811	1.827 (0.537–6.215)	0.334
16,000–49,000	0.671 (0.289–1.556)	0.353	1.179 (0.373–3.73)	0.78
50,000–99,999	0.748 (0.315–1.775)	0.51	0.921 (0.273–3.101)	0.894
Area Deprivation Index	0.703 (0.385–1.282)	0.25	1.259 (0.563–2.816)	0.576
Education level (vs college graduate)				
Some college or technical school, high school graduate, some high school, elementary school	1.135 (0.564–2.281)	1.135	1.001 (0.42–2.385)	0.998
Employment (vs employed)				
Unemployed	1.436 (0.782–2.636)	0.243	1.399 (0.648–3.02)	0.393
Receipt of annual screening mammograms after age of 40 (vs yes)				
No	Not applicable	Not applicable	0.487 (0.225–1.055)	0.068

aOR, adjusted odds ratio; CI, confidence interval

### EEDS and Stage at Diagnosis

The EEDS questions that were statistically significant for an association with late-stage breast cancer at diagnosis were “You receive poorer service than other people at restaurants or stores” (patients with late-stage vs early-stage cancer: less than once a year [18.5 % vs 15.3 %], a few times a year: [18.5 % vs 5.9 %], at least a few times a month [7.4 % vs 5.9 %]; *p* = 0.009); “People act as if they are afraid of you” (patients with late-stage vs early-stage cancer: less than once a year [9.3 % vs 5.9 %], a few times a year [5.6 % vs 2.4 %], at least a few times a month [9.3 % vs 2.4 %]; *p* = 0.026); “You are called names or insulted” (patients with late-stage vs early-stage cancer: less than once a year [14.8 % vs 8.3 %], a few times a year [5.6 % vs 1.7 %], at least a few times a month [9.3 % vs 3.1 %]; *p* = 0.015); “You are threatened or harassed” (patients with late-stage vs early-stage cancer: a few times a year [9.3 % vs 1.4 %]; *p* = 0.008); and “You are followed around in stores” (patients with late-stage vs early-stage cancer: less than once a year [9.3 % vs 5.9 %], a few times a year [11.1 % vs 3.5 %], at least a few times a month [3.7 % vs 1.7 %]; *p* = 0.042 (Table 2).

Multivariable logistic regression showed that a high level of perceived discrimination was associated with late-stage breast cancer at diagnosis (aOR, 1.062; 95 % CI, 1.009–1.118; *p* = 0.022) after control for individual- and contextual-level factors (Table 3). Additionally, the patients of Hispanic ethnicity were less likely to present with late-stage disease (aOR, 0.326; 95 % CI, 0.118–0.898; *p* = 0.03).

### EEDS and Patient–Provider Mistrust

Cronbach’s alpha was 0.89 for the mistrust survey of our patient population, indicating strong survey reliability. Higher perceived discrimination was negatively correlated with multiple measures of trust in the patient–provider relationship. Higher EEDS score negatively correlated with “You understand everything your doctor tells you” (*r* = –0.189; 95 % CI, –0.289 to –0.084; *p* < 0.001), “You feel comfortable telling your doctor when you don’t understand something” (*r* = –0.112; 95 % CI, –0.215 to –0.006; *p* = 0.033), “You feel comfortable telling your doctor when you don’t want a test or procedure” (*r* = –0.139; 95 % CI, –0.241 to –0.033; *p* = 0.008), “You believe your doctor has your best interests at heart” (*r* = –0.228; 95 % CI, –0.325 to –0.125; *p* < 0.001), and “You trust your doctor” (*r* = –0.168; 95 % CI, –0.271 to –0.061; *p* = 0.002) (Table 4).

**Table 4:** Multivariable analysis of correlation between perceived discrimination and patient–provider mistrust

	*r* (correlation coefficient) with EEDS	95 % CI	*p* Value
You understand everything your doctor tells you	− 0.189	− 0.289 to − 0.084	< 0.001
You feel comfortable asking your doctor questions	− 0.096	− 0.199 to 0.01	0.068
You feel comfortable telling your doctor when you don't understand something	− 0.112	− 0.215 to − 0.006	0.033
You feel comfortable telling your doctor when you don't want a test or procedure	− 0.139	− 0.241 to − 0.033	0.008
You believe your doctor has your best interests at heart	− 0.228	− 0.325 to − 0.125	< 0.001
You trust your doctor	− 0.168	− 0.271 to − 0.061	0.002

CI, confidence interval

### Specific Experiences of Discrimination

We found that 57.0 % (*n* = 195) of the patients reported at least one experience of discrimination, based on an EEDS score of at least 1. For these patients, an additional question appeared at the end of the survey asking, “What do you think is the main reason for these experiences,” in relation to the EEDS scale. Of these patients, 111 (32.5 %) responded to this additional question. The most common reason of perceived discrimination was race (*n* = 30, 27 %), followed by the patient’s ancestry or national origin (*n* = 22, 19.8 %) and the patient’s gender (*n* = 17, 15.3 %) (Table 5).

**Table 5:** Descriptive statistics of patient’s response to “What do you think is the main reason for these experiences?”

Reason	Frequency (%)
Your race	30 (27)
Your ancestry or national origins	22 (19.8)
Your gender	17 (15.3)
Your age	11 (9.9)
Your education or income level	9 (8.1)
Your shade of skin color	8 (7.2)
Some other aspects of your physical appearance	8 (7.2)
Your weight	3 (2.7)
Your religion	1 (0.9)
Your height	1 (0.9)
A physical deformity	1 (0.9)

## Discussion

This prospective cohort study of women with newly diagnosed breast cancer treated at an National Cancer Institute (NCI)-designated cancer center and a sister safety-net hospital found that higher perceived discrimination is associated with decreased odds of screening mammography utilization and increased odds of a late-stage diagnosis of breast cancer independent of individual sociodemographic factors and neighborhood-level social determinants of health. Additionally, patients with higher perceived discrimination also reported less trust in their providers, suggesting discrimination also may affect patient–provider relationships, which can contribute to late-stage disease at diagnosis through lack of screening or routine medical attention.

However, to our knowledge, we are the first to associate perceived discrimination with lack of screening mammography and late-stage of breast cancer at diagnosis within the same study and cohort. Our findings are consistent with studies that have shown relationships of discrimination with increased incidence and decreased screening behaviors. Among breast cancer patients, perceived discrimination has been associated with negative physical and psychological well-being and delays in treatment initiation in racial and ethnic minority populations.^10,16^ Even before diagnosis, perceived discrimination has been associated with decreased likelihood of preventive health screening such as screening mammography, although evidence is mixed.^17–19^

Interestingly, those in our study who reported discrimination felt that the main reasons for experiencing discrimination were ancestry, race, and gender. Other studies have shown that minority women perceiving discrimination were less likely to undergo both breast and colorectal cancer screening than their counterparts not perceiving discrimination, whereas women reporting discrimination due to reasons besides racial profiling were less likely to undergo Pap smear, clinical breast exam, and mammography screening.^17,19^

Our findings strengthen the literature by demonstrating that perceived discrimination was associated not only with screening mammography utilization but also with late-stage disease at diagnosis. The reasons for this relationship are multifactorial. Higher levels of perceived discrimination may delay the search for medical attention, ultimately leading to delayed diagnosis and subsequently late-stage disease at diagnosis. In addition to patients presenting with late-stage of disease at diagnosis, delays in diagnosis have an impact on times to surgery, chemotherapy, and radiotherapy, which may ultimately affect survival.^22^

Beyond our finding that perceived discrimination was associated with late-stage at presentation, we found that a higher level of perceived discrimination also strongly correlated with provider mistrust. This is consistent with the previous literature including a study by Sutton et al.,^10^ which found that black women with less trust in their provider were more likely to report experiences of discrimination. Furthermore, findings have shown perceived discrimination to be associated with more mistrust in the patient–provider relationship and reduced perceived quality of care received. In a study conducted in California by Bazargan et al.,^8^ perceived discrimination due to income and insurance status was associated with 98 % higher odds of patients reporting medical mistrust, whereas perceived discrimination due to race, ethnicity, and language spoken was associated with 25 % increased odds of patients reporting mistrust. In another study examining the effect of mistrust on receipt of adjuvant therapy for breast cancer patients, researchers found that patients who reported increased feelings of mistrust were less likely to undergo recommended adjuvant therapy following surgery despite their belief in its benefits.^9^

Our findings showed a direct negative correlation between degree of perceived discrimination and our trust measure, with five of six measures of trust significantly lower for those who experienced higher perceived discrimination. Notably, our findings showed that patients reporting discrimination were less likely to agree with “You believe your doctor has your best interests at heart” and “You trust your doctor.” This supports prior literature by Gonzales et al, in which 56 % of patients who reported discrimination and 50 % who reported clinician mistrust were less likely to report perceived excellence of care received.^23^ Combined, our results further highlight the importance of addressing interpersonal, sociodemographic, and cultural factors that exacerbate or contribute to perceived discrimination and medical mistrust.

Beyond individual-level perceived discrimination and provider mistrust, it is important to acknowledge the upstream structural determinants of health that influence individuals. Previous studies using measures of racial residential segregation have found associations between segregation because of red-lining and breast cancer outcome disparities including late-stage diagnosis, more aggressive tumor subtypes, and shorter survival.^1,3,24^ Red-lining is a discriminatory practice dating back to the 1930s in which services are withheld from customers who reside in neighborhoods classified as “hazardous” to investment. These neighborhoods have significant numbers of racial and ethnic minorities as well as low-income residents.^13^ Studies have shown that women living in historically red-lined neighborhoods as a result of structural racism present with late-stage breast cancer at diagnosis.^1^ The reasons why women residing in these neighborhoods present with late-stage disease likely include individual-level factors such as lack of insurance and contextual-level factors such as limited access to mammography centers.^1^ These data were an important consideration in our decision also to control for neighborhood-level factors such as the ADI in our model.

This study was not without limitations. Our retrospective analysis of outcomes limited causality, and our regional population may limit generalizability of our findings on a national level. Additionally, we did not include patients with ductal carcinoma *in situ* (DCIS), which may have given a more accurate estimation of screening mammography penetrance.

A strength of this study was its utilization of patient-reported survey data not otherwise available via electronic medical record review or in national databases. However, patient-reported surveys are subject to response and recall bias. Our patient population in Miami-Dade County is also extremely diverse in terms of racial, ethnic, and socioeconomic composition. Although our study was limited as a two-center study, the racial and ethnic and economic diversity of our population treated at the NCI-designated cancer center and sister safety-net hospital allowed for robust examination of racialized economic segregation. The patients treated at each hospital were seen by the same physicians who work in both hospital systems, limiting the influence of physician bias or access to certain physicians having an impact on a patient’s perspective.

## Conclusions

Overall, we found that a higher level of perceived discrimination, through the lens of the EEDS, is associated with decreased breast cancer screening mammography utilization and late-stage disease at diagnosis. This may be due to delays in seeking medical attention due to perceptions of discrimination, which lead to delays in diagnosis, resulting in a late stage of disease at diagnosis. In addition, our finding that mistrust of one’s provider also was associated with higher perceived discrimination may further contribute to delays in seeking care. To reduce racial health disparities, special efforts should be made to reach women with a high level of perceived discrimination because this may be a risk factor for lack of screening mammography and late-stage presentation, which ultimately have an impact on breast cancer survival.
