# Correction: Associations Between Perceived Discrimination, Screening Mammography, and Breast Cancer Stage at Diagnosis: A Prospective Cohort Analysis

**DOI:** 10.1245/s10434-024-16009-x

**Published:** 2024-08-05

**Authors:** Alexandra E. Hernandez, Peter A. Borowsky, Maya Lubarsky, Carin Carroll, Seraphina Choi, Susan Kesmodel, Michael Antoni, Neha Goel

**Affiliations:** 1 Division of Surgical Oncology, Department of Surgery, Sylvester Comprehensive Cancer Center, University of Miami Miller School of Medicine, Miami, FL USA; 2 Sylvester Comprehensive Cancer Center, University of Miami, Miami, FL USA; 3 University of Miami Miller School of Medicine, Miami, FL USA; 4 Department of Human Genetics, University of Miami Miller School of Medicine, Miami, FL USA; 5 Department of Psychology, University of Miami Miller School of Medicine, Miami, FL USA

**Correction to: Annals of Surgical Oncology** 10.1245/s10434-024-15757-0

In the original online version of this article, Carin Carroll's last name was misspelled. The original article was corrected.

